# Reply to: Behavior of lung ultrasound findings during spontaneous breathing trial

**DOI:** 10.5935/0103-507X.20180021

**Published:** 2018

**Authors:** Ana Carolina Peçanha Antonio, Cassiano Teixeira

**Affiliations:** Unidade de Terapia Intensiva Adulto, Hospital Moinhos de Vento - Porto Alegre (RS), Brasil.; Unidade de Terapia Intensiva Adulto, Hospital de Clínicas de Porto Alegre- Porto Alegre (RS), Brasil.

First, we would like to thank Drs Blanco and Esquinas for their valuable comments. Indeed, bedside ultrasound became a foremost tool in daily intensive care unit management. International guidelines provide compelling evidence of its benefits and required skills.^(1)^

We fully agree that B-lines are not always pathological. Lichtenstein et al. reported that 28% the healthy subjects in their sample exhibited comet-tail artifacts confined to the last intercostal space above the diaphragm.^(2)^ It should be noted that individuals in our study^(3)^ had in average six days on mechanical ventilation - and, thus, on supine position - before the lung ultrasound analysis. It is not surprising that more than half of our sample already exhibited B and C-lines in lower posterior lung regions, preceding spontaneous breathing trial (SBT), as shown on figure 1.^(3)^ Variables degrees of lung deaeration in dependent areas are likely to represent effects of gravity, perhaps worsened by fluid overload,^(4)^ infectious or inflammatory injury.^(5)^

We were not able to determine the clinical significance of the above findings, however the lack of variation during the spontaneous breathing trial led us to believe that anterior chest zones were sensitive enough to show physiological changes during weaning from mechanical ventilation. As the procedure took up to 120 minutes, the emergence of B-lines during such a short period suggested transudative mechanism, significantly demonstrated for subjects who failed SBT. More or less severe alveolar hydrostatic edema, or even exudative fluid underlying the so-called B-pattern might have played a role, particularly in critically ill subjects who failed to pass SBT. Still, this group showed a significant increase of B-predominance by the end of SBT (Table 2).^(3)^

The performance of B-predominance according to weaning groups on predicting clinical endpoints was notably poor, as shown on table 3.^(3)^ Shortcomings of our investigation were also disclosed. We certainly cannot rule out the usefulness of echocardiography, and pleural and vein ultrasound examinations during weaning from mechanical ventilation and in many critical and emergency scenarios. However, we cannot completely agree that intensivists could accurately perform such assessments without consuming too much time. Although the Blue Protocol is based on six-region scans,^(6)^ the authors designated A and B profiles based on anterior predominance of corresponding lines.

We completely agree that there is a compelling need to explore the full potential of lung ultrasound.

*Ana Carolina Peçanha Antonio* *Adult Intensive Care Unit, Hospital Moinhos de Vento - Porto Alegre (RS),
Brazil.* *Adult Intensive Care Unit, Hospital de Clínicas de Porto Alegre-
Porto Alegre (RS), Brazil.* *Cassiano Teixeira* *Adult Intensive Care Unit, Hospital Moinhos de Vento - Porto Alegre (RS),
Brazil.* *Adult Intensive Care Unit, Hospital de Clínicas de Porto Alegre-
Porto Alegre (RS), Brazil.*
